# Effect of PVOH/PLA + Wax Coatings on Physical and Functional Properties of Biodegradable Food Packaging Films

**DOI:** 10.3390/polym14050935

**Published:** 2022-02-25

**Authors:** Annalisa Apicella, Antonio Barbato, Emilia Garofalo, Loredana Incarnato, Paola Scarfato

**Affiliations:** Department of Industrial Engineering, University of Salerno, Via Giovanni Paolo II, 132, 84084 Fisciano, SA, Italy; abarbato@unisa.it (A.B.); egarofalo@unisa.it (E.G.); lincarnato@unisa.it (L.I.); pscarfato@unisa.it (P.S.)

**Keywords:** biodegradable film, polylactic acid (PLA), polyvinyl alcohol (PVOH), oxygen barrier, multifunctional packaging, wax, double coating, water resistance

## Abstract

Biodegradable polymers suffer from inherent performance limitations that severely limit their practical application. Their functionalization by coating technology is a promising strategy to significantly improve their physical properties for food packaging. In this study, we investigated the double coating technique to produce multifunctional, high barrier and heat-sealable biodegradable films. The systems consisted of a web layer, made of poly(lactide) (PLA) and poly(butylene-adipate-co-terephthalate) (PBAT), which was first coated with a poly(vinyl) alcohol based layer, providing high barrier, and then with a second layer of PLA + ethylene-bis-stereamide (EBS) wax (from 0 to 20%), to provide sealability and improve moisture resistance. The films were fully characterized in terms of chemical, thermal, morphological, surface and functional properties. The deposition of the PVOH coating alone, with a thickness of 5 μm, led to a decrease in the oxygen transmission rate from 2200 cm^3^/m^2^ d bar, for the neat substrate (thickness of 22 μm), to 8.14 cm^3^/m^2^ d bar (thickness of 27 μm). The deposition of the second PLA layer did not affect the barrier properties but provided heat sealability, with a maximum bonding strength equal to 6.53 N/25 mm. The EBS wax incorporation into the PLA slightly increased the surface hydrophobicity, since the water contact angle passed from 65.4°, for the neat polylactide layer, to 71° for the 20% wax concentration. With respect to the substrate, the double-coated films exhibited increased stiffness, with an elastic modulus ca. three times higher, and a reduced elongation at break, which, however still remained above 75%. Overall, the developed double-coated films exhibited performances comparable to those of the most common synthetic polymer films used in the packaging industry, underlining their suitability for the packaging of sensitive foods with high O_2_-barrier requirements.

## 1. Introduction

Packaging, including flexible films and rigid containers, represents the largest single market for the consumption of plastics, with 23 million tons per year (and 92 million tons expected in 2050). In particular, flexible film production is one of the fastest-growing sectors in the packaging industry, with a compound annual growth rate CAGR of 5% [1,2,3].

This represents a major source of environmental pollution. The use of biodegradable and/or compostable materials aims to minimize the environmental impact induced by post-consumer synthetic plastic waste. Even with promising trends for applicability thanks to their undoubted ecological advantages, biodegradable polymers present some disadvantages. Compared to conventional polymers, biodegradable polymers generally have scarce gas and vapor barrier properties, especially in humid environments, poor mechanical performance, especially in terms of ductility, and low thermal stability [4].

The oxygen permeability of biodegradable polymers that are of major interest for commercial applications, i.e., PLA and its blends with PBAT, polycaprolactone (PCL) and starch, is at least 20 times higher than that of PET, and even more than two orders of magnitude larger than high-performance conventional, fossil-based materials [5,6,7,8,9,10,11,12,13,14,15]. These limitations are particularly critical in the case of flexible packaging, whose thickness is very small, and severely limit its application to foods with low moisture content and short shelf-life [11,16]. Therefore, it is necessary to implement appropriate functionalization strategies with the purpose of expanding the application field of biodegradable plastics.

In this regard, the deposition of functional bio-based coatings is a promising approach to improving the surface characteristics of substrates (i.e., adhesion, wettability, water repellence, anti-corrosion, antioxidant, antimicrobial and gas barrier properties) without compromising the biodegradable and/or recyclable features [17]). Polymeric coatings based on chitin nanofibrils, chitosan or PLA-based composites demonstrated their effectiveness for antimicrobial packaging [18,19,20,21,22,23]; hydrophobic properties can be imparted by including chitin or waxes [24,25,26] or by acrylic-modified crosslinkable chitosan nanocoatings [27]; among efficient oxygen-barrier layers, crosslinked proteins [28,29,30,31,32], cellulose nanocrystals [33,34,35] and poly(vinyl alcohol) [36,37,38,39,40] have recently received attention due to their excellent barrier performance.

Polyvinyl-alcohol (PVOH), in particular, is one of the few completely biodegradable synthetic polymers showing multiple desirable features: it features good biocompatibility, excellent transparency, gas/aroma barrier, film-forming ability and chemical resistance [41,42,43]. It is also approved by the US Food and Drugs Administration (FDA) and the European Medicines Agency (EMA) for food contact applications [44,45]. However, PVOH has limitations as an individual packaging material due to its high water sensitivity, which strongly limits its application in foods with high water activity [46,47].

In this study, we investigated the double-coating technique to produce three-layer, multifunctional films with a high barrier, heat-sealable properties and improved moisture resistance.

The web layer, a ductile and tear-resistant blown film made of PLA and PBAT, was coated with a first layer based on an ethylene-modified PVOH, providing a high barrier. Next, a second coating layer made of an amorphous grade of PLA + EBS wax at different loads (from 0 to 20%) was spread to provide sealability [48] and to protect the water-sensitive PVOH layer, giving moisture resistance to the structure. To the best of the authors’ knowledge, this was the first time that the combined use of PVOH and PLA + wax for double coatings was proposed to achieve this target. Moreover, very few studies focused on the development of double- or multiple-coated films [36]; those that did mainly did so through the layer-by-layer (LBL) assembly technique [49], mostly for medical applications [50], electrically conductive coatings [51] and devices for drug delivery [52]. The produced films were characterized by differential scanning calorimetry (DSC) and infrared spectroscopy (ATR FT-IR) to investigate their inherent chemical and thermal properties, as well as possible interactions between the micronized wax and the PLA matrix. The uniformity of the layers’ thickness and the quality of the interlayer adhesion were evaluated by SEM analyses. Next, the surface wettability, the oxygen permeability, and the mechanical and heat-sealable properties were quantified to evaluate the potential of the developed solution for biodegradable packaging with superior barrier and water resistance properties.

## 2. Experiment

### 2.1. Materials

**Figure 1:**
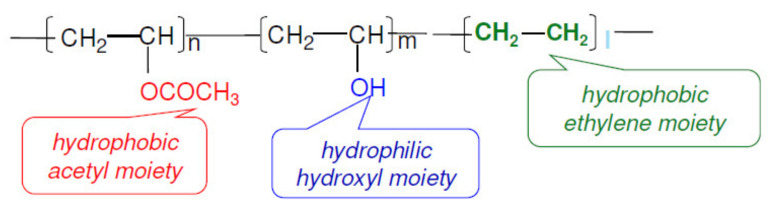
Chemical structure of Exceval™ AQ-4104.

A commercial PLA/PBAT blend, under the trade name Bioter™ (Euromaster S.p.a, Pistoia, Italy), was selected for the production of the blown web layer. Exceval AQ-4104 is an ethylene-modified polyvinyl alcohol, as displayed in Figure 1. It is fully hydrolyzed, water-soluble, chlorine-free and FDA-approved for food contact. According to the producer’s technical information, the polymer, hereafter referred to as m-PVOH, maintains constant gas barrier properties in a wide relative humidity range, from dry conditions to ca. 60% [53].

PLA4060D (amorphous, d-isomer content = 12 wt%, Mw ~190,000 g/mol, specific gravity = 1.24 g/cm^3^) was supplied by Natureworks (Minnetonka, MN, USA). Ethylene-bis-stearamide (EBS) wax X2010M, with a particle size of 98% < 10 μm, density of 0.98–1 g/cm^3^, and drop point of 142–151 °C, was generously provided by Deurex (Elsterau, Germany). The wax derived from the revaluation of sugar cane wastes and was approved for direct food contact. All the solvents used were analytical-grade.

### 2.2. Production of the Multilayer Films

Bioter granules were dried at 70 °C for 14 h prior to processing. The substrate film, with a thickness of 22 ± 2 µm, was produced by a GIMAC blown film plant, equipped with a single-screw extruder (D = 12 mm, L/D = 24). The thermal profile was set between 190 °C and 160 °C, with a screw speed of 50 rpm and a collection speed of 3 m/min.

Double-coating procedure was conducted using a laboratory bar coating technique, which can be further easily scaled up to a factory level through gravure roll coater.

The m-PVOH-coating solution was prepared by dissolving the polymer in deionized water with a mass ratio of 10:90; this optimized composition was suggested by the producer technical data sheets [53]. The mixture was then heated up to 95 °C and stirred until complete dissolution of the polymer. Isopropanol was added as anti-foaming agent to the coating mixture, in a mass percentage equal to 10% with respect to the m-PVOH weight. The solution was cooled down to ambient temperature and spread on the Biofilm web, along the machine direction, by means of a K Hand Coater (RK, Printocoat Instruments Ltd., Litlington, UK), equipped with stainless steel closed wound a rod with a wire diameter equal to 0.64 mm, yielding final coatings with comparable thickness equal to 5 ± 1 µm. Coated films were subjected to a drying step at 120 °C for 3 min in oven and stored overnight at room conditions before the second coating layer deposition.

The PLA coating solution was prepared according to the method described by Apicella et al. (2019). The polymer was dissolved in acetone with mass ratio 20:80 and wax was added at 0%, 5% 10% and 20% w/w_PLA_. The mixture was spread on the dried m-PVOH-coating layer, yielding final coatings whose average coating layer thickness was between 6 and 8 µm. Table 1 summarizes the list of the prepared films.

### 2.3. ATR-FTIR Analyses

Fourier transform-infrared spectrometry was carried out by means of a Nicolet 600 FT-IR spectrophotometer (Thermo Scientific, Waltham, MA, USA) in attenuated total-reflection (ATR) mode. The spectra were collected at a resolution of 4 cm^−1^, averaging over 54 scans in the range of 650–4000 cm^−1^. Normalization and peak integration were performed by Omnic software (ver. 6.2).

### 2.4. Thermal Characterization

Thermograms were obtained on a Differential Scanning Calorimeter (DSC) model 822 (Mettler Toledo, Columbus, OH, USA). The temperature range studied was 25 to 200 °C, with heating/cooling rate equal to 10 °C/min, under a nitrogen gas flow equal to 50 mL/min.

### 2.5. Scanning Electron Microscopy (SEM)

Microstructure of the cross section of the films was characterized by SEM analyses. The film samples were cryofractured in liquid nitrogen normally to the coating direction, and then sputter-coated with gold (Agar Auto Sputter Coater model 108A, Stansted, UK) at 30 mA for 160 s and analyzed by scanning electron microscope model LEO 1525 (Carl Zeiss SMT AG, Oberkochen, Germany).

### 2.6. Oxygen Transmission Rate Measurements

Oxygen transmission rate (OTR) measurements were performed by means of a gas permeabilimeter (GDP-C, Brugger, Munich, Germany). The tests were carried out in triple at 23 °C and 0% R.H., under pressure difference of oxygen equal to 1 bar, with the oxygen flow rate of 80 mL/min, according to ISO 15105-1.

In order to obtain the oxygen permeability values (P_O2_) of the Biofilm and Biofilm/m-PVOH films, the oxygen transmission rates were multiplied by the film thicknesses. This latter value is a mixed value of different material layers, and was calculated assuming as major simplification that the multilayer film behave as an homogeneous material [5]. Resulting oxygen permeability of the Biofilm/m-PVOH coated film was used for further calculations of the permeability of the single m-PVOH layer, assuming the film as two-layer structure. The following equations can be used [5]:

(1) $$\frac{x_{tot}}{P_{tot}}=\frac{x_{1}}{P_{1}}+\frac{x_{2}}{P_{2}}$$

(2) $$\frac{1}{Q_{tot}}=\sum \frac{x_{i}}{P_{i}}=\frac{1}{Q_{1}}+\frac{1}{Q_{2}}+\ldots$$

where x and P represent the thickness and the permeability of each layer i. Subscript “1” stands for the Biofilm substrate, subscript “2” for the m-PVOH-coating and subscript “*tot*” for the whole multilayer structure.

### 2.7. Evaluation of Wettability, Surface Energies and the Work of Adhesion

Films wettability was evaluated by static contact angle measurements using a First Ten Angstrom Analyzer System 32.0 model FTA 1000 (First Ten Angstroms, Inc., Portsmouth, VA, USA), according to the standard test method, ASTM D5946. The drop volume was taken within the range where the contact angle did not change with the variation in the volume (2 ± 0.5 µL). Each reported value of the θ angle is the average of at least ten replicate measurements.

Surface-free energies (SFEs) of the substrate and coating surfaces were estimated by applying both the Owens–Wendt (OW) geometric mean equation [54] and Van Oss–Chaudhury–Good (VCG) acid-base theory [55,56]. According to the first approach, the total surface energy is the sum of dispersive (*γ_s_^d^*) and polar (*γ_s_^p^*) components. In the second approach, it is the sum of a Lifshitz–van der Waals (*γ_s_^LW^*) component and a Lewis acid-base (*γ_s_^AB^*) component, $\gamma_{s}^{AB}=2\sqrt{\gamma_{s}^{+}\gamma_{s}^{-}}$, which further includes an electron acceptor (*γ_s_*^+^) and an electron donor (*γ_s_*^−^).

Distilled water (H_2_O) and diiodomethane (DM) were used as testing liquids for Owens–Wendt method, while H_2_O, DM and ethylene glycol (EG) were used for Van Oss–Chaudhury–Good model. The SE components for the testing liquids can be found elsewhere [18,57].

The work of adhesion *W_a_* is defined as the reversible thermodynamic work required to separate the interface from the equilibrium state of two phases to a separation distance of infinity [58] and can be calculated by Dupre equation:

(3) $$\;W_{a}=\gamma_{S}+\gamma_{L}-\gamma_{SL}$$

where *γ_L_* is the surface energy (tension) of the liquid phase, *γ_S_* is the surface energy of the solid phase and *γ_SL_* is the interfacial energy. As the surface energies of the substrate and coating layers can be calculated by the OW and VCG approaches, the work of adhesion can be evaluated using the following equations:

(4) $$W_{a}^{g}=2\left(\sqrt{\gamma_{S}^{d}\gamma_{L}^{d}}+\sqrt{\gamma_{S}^{p}\gamma_{L}^{p}}\right)$$

(5) $$W_{a}^{ab}=2\left(\sqrt{\gamma_{S}^{LW}\gamma_{L}^{LW}}+\sqrt{\gamma_{S}^{+}\gamma_{L}^{-}}+\sqrt{\gamma_{S}^{-}\gamma_{L}^{+}}\right)$$

where $W_{a}^{g}$ and $W_{a}^{ab}$ are the work of adhesion following the OW and VCG approaches, respectively. According to Equation (3), a high work of adhesion requires not only high surface energies of both the substrate and the coating layers, but also low interfacial energy. Low interfacial energy values indicate high interfacial substrate–coating or coating–coating compatibility.

### 2.8. Evaluation of the Seal Strength

In order to evaluate the seal strength of the films, delamination tests were carried out by SANS dynamometer (mod. CMT 6000 by MTS, Shenzhen, China), equipped with a 100 N load cell, in accordance with the standards ASTM F88-00 and ASTM F2029-00. The film samples were cut in strips of 200 × 25 mm^2^ and sealed using a heat-sealing machine (mod. HSG-C, Brugger, Munich, Germany). Specimens were sealed at different temperatures, between 75 °C and 105 °C; these temperatures corresponded to the seal initiation temperature and the temperature above which the film suffers excessive distortion and shrinkage, respectively; the dwell time was set equal to 1 s and the clamp force was set at 690 N. Next, according to the ASTM F88-00 standard, they were conditioned at 23 °C and 50 ± 5% R.H. for 48 h prior to testing. The bonding strength was evaluated in tensile mode, fixing a crosshead speed equal to 250 mm/min until seal failure. For each sample type, at least ten measurements were performed to assess the reproducibility of the results.

### 2.9. Tensile Tests

Tensile tests were performed on a SANS testing machine (model CMT 6000 by MTS, Shenzhen, China), equipped with a 100 N load cell. The tests were carried out according to the standard ASTM D 882-91. Film specimens were cut with a rectangular geometry (12.7 × 80 mm^2^) along the coating direction, conditioned for 48 h at 23 °C and 50 ± 5% R.H. and tested in the same conditions. The results are expressed in terms of elastic modulus (E), tensile strength (σ_b_) and percentage elongation at break (ε_b_) for all the tested films. The crosshead speed of the test was kept at 3 mm/min for the duration of each test for the evaluation of elastic modulus (E) and 300 mm/min for the assessment of tensile strength (σ_b_) and percentage elongation at break (ε_b_). For multilayer structures, the tensile parameters are mixed values of different material layers, and were calculated assuming, as a major simplification, that multilayer films behave as homogeneous materials.

### 2.10. Statistical Analysis methodology

The results are reported as the arithmetic mean, or average, of at least ten replicate measurements, unless otherwise reported. The square root of the variance (i.e., the standard deviation), was used to describe the dispersion of the set of values around the mean. 

## 3. Results

### 3.1. FTIR-ATR Analysis

FTIR spectroscopy was carried out to determine the characteristics of the film matrices, as well as possible changes in the intra- and intermolecular interactions due to the coating production. Due to the limited penetration depth of infrared radiation into the sample in the ATR-FT-IR measurement geometry, the spectra were collected on the coating layers, i.e., m-PVOH and PLA, at different percentages of wax, deposited on the substrate.

**Figure 2:**
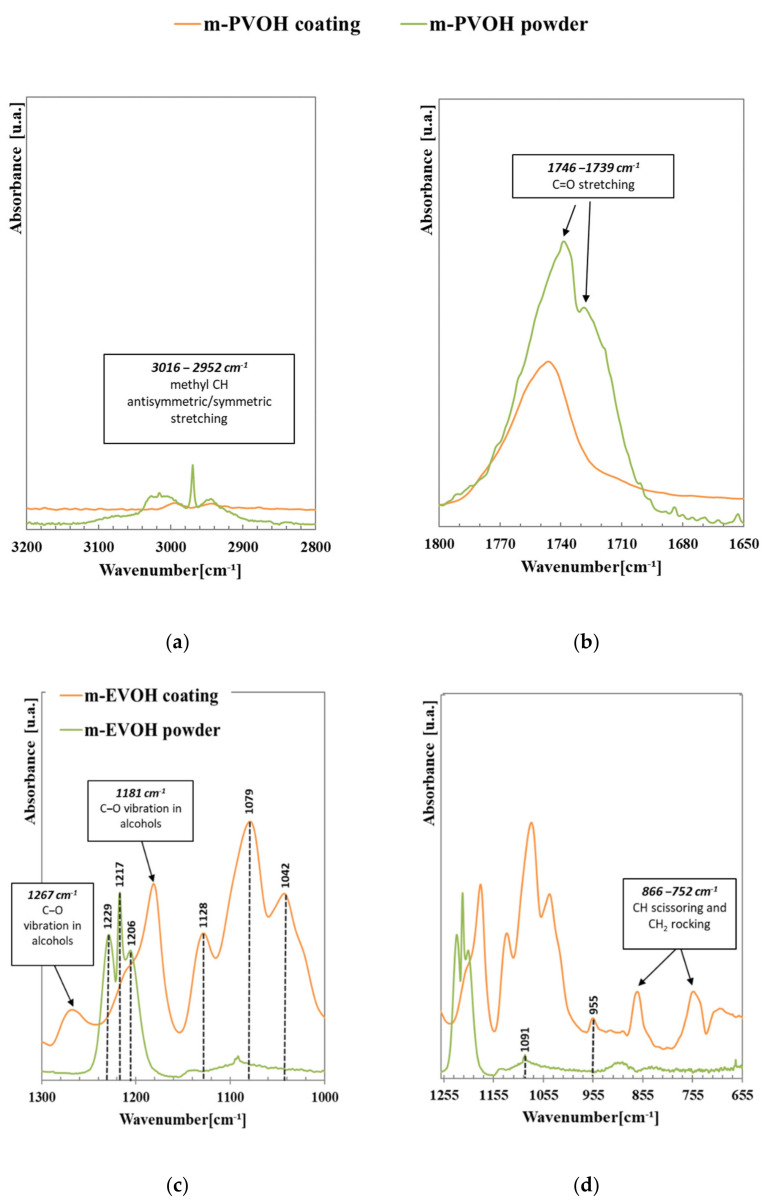
(**a**–**d**) ATR-FTIR spectra magnification of m-PVOH powder (green line) and m-PVOH thin coating layer (red line).

Figure 2 shows the FTIR-ATR spectrum of the m-PVOH-coating layer, in comparison with that of the original m-PVOH powder. The graph highlights multiple relevant differences between the samples, which indicate that the coating procedure modified the polymer’s structural organization. The ATR-FTIR trace of the m-PVOH powder shows the following main characteristic absorptions: the complex multiple band between 3016–2952 cm^−1^ is the superimposition of O–H (hydrogen bonded, intramolecular) and C–H (methyl) stretching; 1428  cm^−1^ (CH_2_ bending); 1739 and 1746 cm^−1^ (C=O stretching); 1229 cm^−1^, 1217 cm^−1^ and 1206 cm^−1^ (COC, C–O and C–C stretching); and 1091  cm^−1^ (C–O stretching). Most of these signals modified shape and intensity and other new signals arose in the ATR-FTIR trace of the m-PVOH-coating layer. The main changes were observed in the O–H and C=O absorption bands, which reduced their intensity, thus indicating a modification to the H-bonding arrangements of the polymer chains; this was accompanied by the appearance of new signals at 1450 cm^−1^ (methyl group) and in the 1150–1050 cm^−1^ region (C–O stretching, secondary alcohol). These findings may be ascribed to the presence in the coating layer of residual isopropanol, used as an anti-foaming agent in the coating solution. Since isopropanol is able to form strong hydrogen bonds with m-PVOH, it can be hypothesized that, in the conditions used for the experiments, isopropanol did not evaporate during the film formation, but remained in the coating. A possible consequence of the polymer’s intramolecular interaction inhibition may have been the formation of an extensive intermolecular H-bond network among the m-PVOH chains.

**Figure 3:**
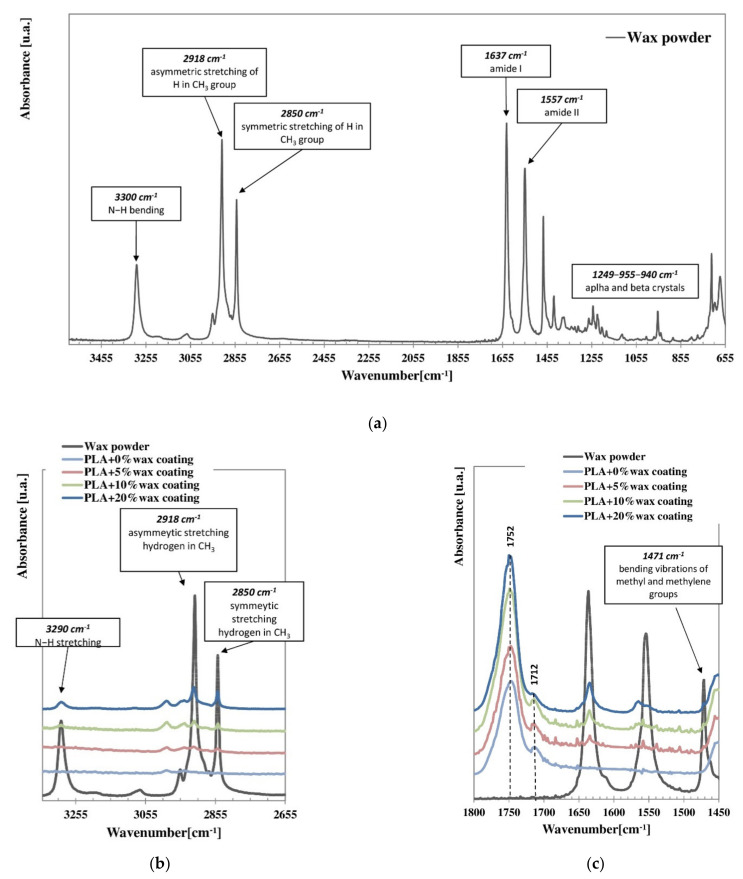
ATR-FTIR spectra of EBS wax (**a**) and of the thin PLA-coating layers loaded at 0, 5, 10 and 20% wax, respectively (**b**,**c**).

Figure 3 displays the FTIR spectrum of the EBS wax powder (a), and the comparison with the spectra of the thin PLA-coating layers at different wax percentages (b, c).

The ATR-FTIR trace of EBS wax powder (Figure 3a) shows the following absorption peaks. The band at 3300 cm^−1^ is due to N–H stretching and those in the regions of 2918 cm^−1^ and 2850 cm^−1^ are ascribed to the asymmetric and symmetric stretching vibration of hydrogen in the methyl group [59]. The peaks at 1637 cm^−1^ and 1557 cm^−1^ correspond to the amide I (C=O stretching) and amide II (mixed vibration of N–H bending and C–N stretching) bands, respectively. The three characteristic bands situated at 1248, 955 and 940 cm^−1^ correspond to the interconversion of crystalline forms of EBS wax: in particular, the band at 955 cm^−1^ is characteristic of the alpha crystalline form, while the appearance of the other two bands is characteristic of the beta form. Their appearance confirms the simultaneous presence of crystals in both alpha and beta forms [60].

The trace of the PLA + 0% wax coating layer (Figure 3b,c) shows all the characteristic absorption peaks of the neat polymer. The main peaks are: 2980 cm^−1^ (C–H, methyl), 1748 cm^−1^ (C=O, ester), 1435 and 1395 cm^−1^ (C–O–H bending) and 1270–1083 cm^−1^ (C–O–C stretching). In addition to these, in the spectra of the PLA coatings with 5, 10 and 20% wax, wax absorption bands also appeared, becoming increasingly intense as the wax content increased. This indicated the production of a physical mixture, with no alteration to the molecular structures of the constituents. However, the spectral features also suggested the occurrence of some degree of chemical interaction between the two phases. In fact, as highlighted in Figure 3c, the PLA C=O ester signal at 1748 cm^−1^, which was highly sensitive to the polymer morphology and crystalline order, changed its shape, with the shoulder lowering at 1712 cm^−1^ and splitting occurring at 1752 cm^−1^, indicating a change in the PLA conformation and specific interactions [61]. Moreover, comparing the amide I and amide II bands of the EBS, whether neat or incorporated into the PLA, which were sensitive to the intramolecular hydrogen bonding state of the C=O and N-H moieties of the wax, a decrease in the amide II/amide I intensity ratios in the blends was observed. This was due to a reduction in the intramolecular H-bonding in favor of the formation of intermolecular associations involving PLA.

### 3.2. Thermal Transition and Crystallinity

DSC measurements were carried out on the thin m-PVOH and PLA-coating layers in order to obtain information on their morphology and to investigate possible changes in thermal transitions due to the coating processing and/or the wax’s incorporation in the PLA matrix.

**Figure 4:**
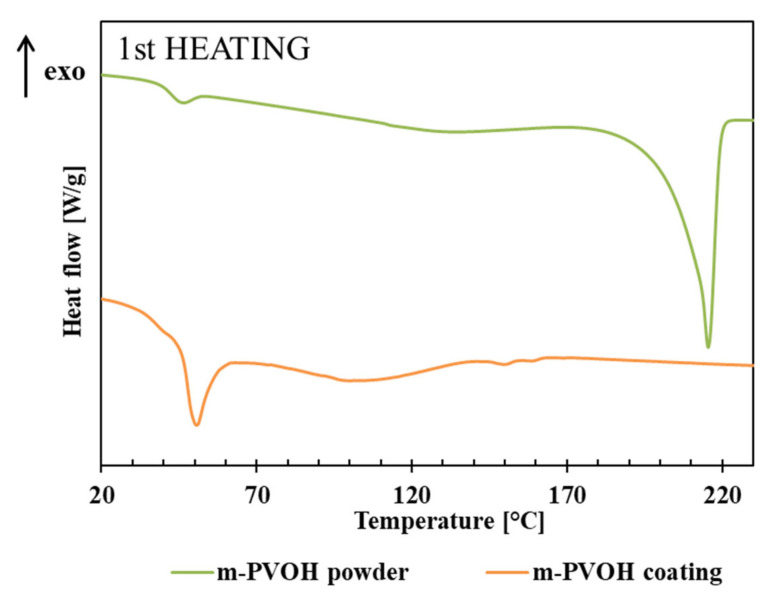
DSC first heating thermogram of m-PVOH powder and m-PVOH thin-coating layer.

The first heating thermograms and thermal parameters related to the first heating and cooling of m-PVOH powder and m-PVOH-coating samples are reported in Figure 4 and Table 2, respectively.

The m-PVOH powder exhibited a glass transition temperature in the first heating scan around 43 °C, lower than the T_g_ values (ca. 70–80 °C) reported in literature for fully hydrolyzed, unmodified PVOH grades [62]. A further slight decrease in T_g_ was obtained in the m-PVOH coating.

Polyvinyl alcohol is widely recognized as a crystalline water-soluble polymer. It is known that the degree of crystallinity varies depending on the degree of hydrolysis and the degree of polymerization. In particular, the influence of the degree of hydrolysis is large, and the higher the degree of hydrolysis, the higher the crystallinity tends to be. Additionally, it is known that the degree of crystallinity changes with external factors, and it is particularly susceptible to the influence of heat treatment [63]. m-PVOH powder exhibits a melting peak at T_m_ equal to 150.7 °C, while it is worth to noting the absence of melting peaks within the m-PVOH-coating film. This behavior is coherent with the results of FTIR analysis, which evidenced the disruption of the m-PVOH intramolecular H-bond arrangements in the coating layer, resulting in significant changes in its structural and morphological state. Other authors also reported the impact on the T_g_, the free volume, and the disruption of the PVOH crystalline phase by increasing the loading amount of plasticizers and additives (alcohols, glycerol, propylene glycol, drugs, etc.) [64,65].

Concerning the melt crystallization behavior, the m-PVOH powder showed a double-shoulder crystallization peak, with Tc around 151 °C. By contrast, the m-PVOH-coating sample was still amorphous and failed to crystallize in the cooling conditions fixed during the DSC scan.

**Figure 5:**
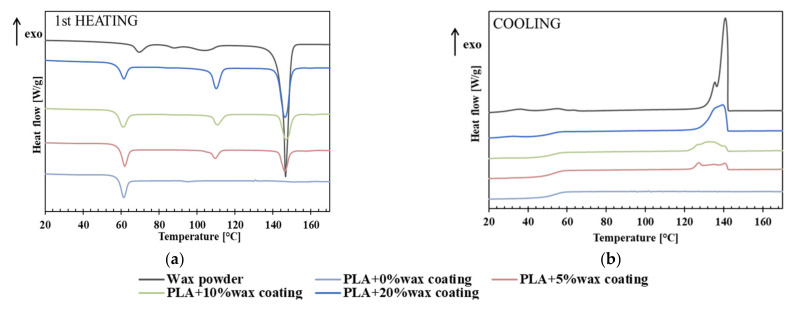
DSC first heating (**a**) and cooling (**b**) thermograms of wax powder and PLA coating layers with 0%, 5%,10% and 20% of wax.

Figure 5 displays the first heating (a) and cooling (b) thermograms for the neat EBS wax powder and the thin PLA-coating layers loaded with 0%, 5%, 10% and 20% of wax. The main thermal parameters related to the first heating and cooling scans are reported in Table 3.

The wax first-heating thermogram (Figure 5a) revealed heterogeneous crystalline domains, consisting of a mixture of alpha and beta crystals; these are typical of EBS, as confirmed by literature data [66]. In particular, at 60 °C, an endothermic event specific to the alpha-to-beta full transition began. Around 80 °C, the EBS wax was found mainly in the beta form, which around 105 °C reverted back to the alpha form. At 120 °C, the EBS was mainly in the alpha form again, and then at around 140 °C the complete melting of the EBS began, in a classic endothermic event [60].

As expected, the PLA + 0% wax coating sample was completely amorphous, and only the PLA glass transition was visible; this transition occurred around 60 °C and was followed by the typical relaxation associated with polymer ageing. At larger concentrations of wax within the PLA layer, wax characteristic melting peaks arose around 110 °C (beta-to-alpha crystal transition) and 146 °C. In particular, an increase in the associated melting enthalpies was detectable by increasing the wax percentage.

The EBS wax also showed polymorphic crystallization peaks during the cooling step (Figure 5b) in all the analyzed samples; in particular, low wax loads (≤10%) in the PLA matrix led to broader crystallization peaks, suggesting the formation of heterogeneous and less-perfect crystals, while at higher wax load (20%), the crystallization peak became sharper. Furthermore, the crystallization enthalpies were increased by increasing the wax percentage.

### 3.3. SEM Analyses

The films’ morphology and the microstructural properties of the multilayer films were investigated trough SEM analyses, as it is known that they affect the final physical, mechanical, barrier and optical properties. In particular, this investigation was useful to understand the quality of interlayer adhesion among the substrate and the coating layers, as well as the wax distribution inside the PLA matrix.

**Figure 6:**
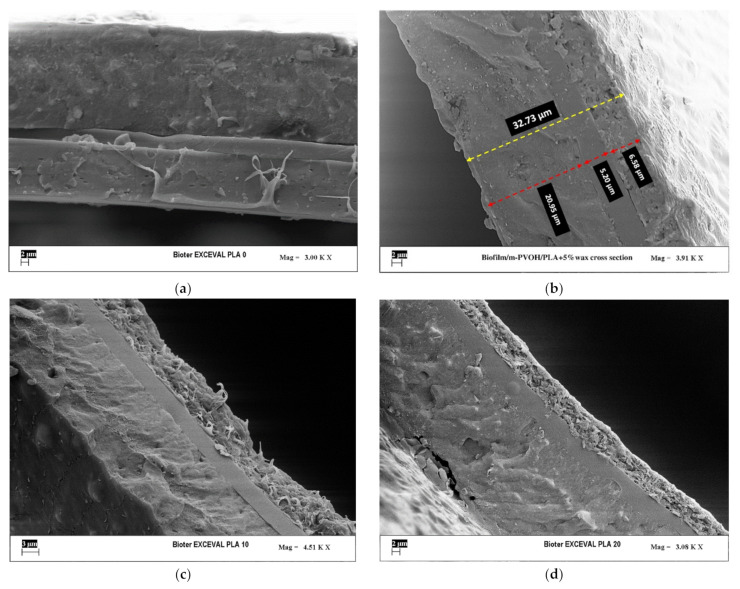
Cross-sectional SEM micrographs, respectively, of: Biofilm/m-PVOH/PLA + 0% wax (**a**), Biofilm/m-PVOH/PLA + 5% wax (**b**), Biofilm/m-PVOH/PLA + 10% wax (**c**), Biofilm/m-PVOH/PLA + 20% wax (**d**) films.

Figure 6 shows the cross-sectional micrograph and surface micrographs of the Biofilm/m-PVOH/PLA + 0% wax (a), Biofilm/m-PVOH/PLA + 5% wax (b), Biofilm/m-PVOH/PLA + 10% wax (c), Biofilm/m-PVOH/PLA + 20% wax (d) films, respectively.

The images of the analyses display the PLA coating layer, in which few small voids are recognizable due to solvent evaporation, and the m-PVOH-coating layer, characterized by an homogeneous morphology, over the Biofilm substrate.

In all the samples, the Biofilm substrate shows a two-phase morphology typical of immiscible systems such as PLA/PBAT, with PLA small globular domains dispersed in the PBAT matrix with quite uniform distribution and average size (<1 micron). Indeed, data from the literature confirm that when PBAT is the dispersed phase, the polymer shows an elongated, fibrous morphology within the PLA matrix, whereas when PBAT is the matrix phase, the PLA morphology turns into globular domains [9]. The size of the PLA domains within the PBAT matrix suggests low interfacial tension and good compatibility between the two constituents, achieved due to the use of additives or compatibilizers [67].

For all the multilayer films, it was possible to observe the obtainment of good inter-layer adhesion, highlighted by the absence of boundary lines or voids among the Biofilm substrate, the m-PVOH and the PLA coating layers, in the whole cross-sectional area. Moreover, the calculation of the thicknesses of both the coating layers underlined a good agreement with the nominal thicknesses, highlighting their good control during the coating process.

It is worth to noting that the wax’s incorporation in the PLA matrix was responsible for clear modifications in the polymer’s morphology, which were rendered more consistent by increasing the wax percentage. Although wax domains were not clearly detectable within the cross-section, the PLA coating surface became more irregular and rougher as the wax percentage increased. This particular morphology does not affect the quality of the interlayer adhesion with the m-PVOH layer; nonetheless it may represent a detrimental factor for the heat seal extent.

### 3.4. Oxygen Barrier Properties

The oxygen barrier is one of the main characteristics required in the design of materials for food packaging applications, since many decomposition reactions depend on the presence of oxygen, which generates a rapid decline in the safety and quality of food products.

To investigate the effects of the system composition on the barrier performance of the samples, OTR tests were carried out. The outcomes are reported in Table 4.

The oxygen transmission rate of the neat Biofilm substrate is comparable to the literature data reported for films based on PBAT/PLA blends [9].

As expected, the addition of a m-PVOH layer strongly decreases the oxygen transmission rate of the Biofilm substrate due to its excellent oxygen barrier properties [38,68]. A three-order-of-magnitude reduction in OTR was registered by comparing the Biofilm/m-PVOH film and the neat substrate (2200 ± 83.6 and 8.14 ± 1.04 cm^3^/m^2^ d bar, respectively). The deposition of the amorphous PLA-based second coating layers did not substantially affect the barrier properties of the films; this was expected, since the oxygen permeability of the amorphous PLA (~2300 cm^3^ mm/m^2^ d bar) is approximately four 4 orders of magnitude higher than that of Biofilm/m-PVOH support film [69].

The oxygen permeability of the single m-PVOH-coating layer was also calculated from the oxygen permeability values (P_O2_) of the Biofilm and Biofilm/m-PVOH films, basing on the simplifying assumption mentioned above, in the “Experiment” section, and according to the Equations (4) and (5), yielding to a value equal to 0.047 cm^3^ mm/m^2^ d bar. Table 5 reports the oxygen permeability of the m-PVOH single-coating layer compared to those of other biodegradable materials [11].

In accordance with the classification proposed by J. Wang et al. [70], the deposition of the m-PVOH-coating layer improves the barrier performance of the substrate, which falls within the range of low-barrier biodegradable polymers (4–40 cm^3^ mm/m^2^ d bar), up to a high barrier (0.040–0.40 cm^3^ mm/m^2^ d bar). It is clear that the classification of the barrier grade is not only function of the permeability performance, but mainly depends on the shelf-life requirements of the target food. In our case, the P_O2_ reduction suggests the possible application of the developed films for the packaging of sensitive foods with high O_2_-barrier requirements, such as fresh meats and cheese [11]; the target food therefore depends on the overall performance of the packaging, including its water vapor permeability.

### 3.5. Evaluation of Wettability, Surface Energies and the Work of Adhesion

Contact angle measurements were carried out to investigate the surface hydrophobicity; moreover, they made it possible to calculate the films’ surface energies and to evaluate the extent of the interlayer adhesion in the coated systems.

The results of the static equilibrium contact angles of the different multilayer surfaces are presented in Table 6. Using these angles, the surface energies of the Biofilm substrate, m-PVOH and PLA-based coating layers were estimated.

According to the Owens–Wendt approach, the neat Biofilm substrate showed highly hydrophobic behavior, with a low polar fraction of surface energy and the lowest polarity value $\left(P_{s}=\gamma_{s}^{p}/\gamma_{s}^{}\right)$, equal to 0.07. Hydrophobicity can be enhanced by some additives, such as cardanol oil [71] and pine resin derivatives [72], by chain extenders, such as Joncryl ADR 4370S [73], or by particular arrangements and conformations of polymer chains [74]. On the other hand, the m-PVOH coating had higher surface energy, with an increased polar fraction and the highest polarity value (equal to 0.43), as a consequence of the number of polar units (–OH and C=O groups) in its structure. The water contact angle value was slightly larger than the values reported in the literature (39–42° for fully hydrolyzed PVOHs [47]), which is ascribable to the presence of ethylenic insertions in the chains. In a similar fashion, the Lewis acid-base component of the m-PVOH surface was much higher than that of the Biofilm, featuring a positive charge on the surface with a higher acid component.

The further deposition of the PLA coating incorporated with EBS wax increased the surface hydrophobicity, as evidenced by the increase in water contact angle and the decrease in polarity values, which was rendered more consistent by increasing the wax concentration. The positive effect of the PLA + wax coating layer deposition on the improvement of the surface hydrophobicity and of the water vapor barrier properties will be further investigated by water vapor permeability tests, which will be object of future study. Furthermore, the addition of wax to PLA determines a decrease in the polar component and, at the same time, no substantial modification of the dispersive component; this effect may be attributed to the presence of a large amount of aliphatic chains, characteristic of EBS, that diminish the polar interactions, and is also reflected in the increase in Lifshitz–van der Waals and base components in the OCG method.

In general, SFEs obtained from the two methods were of the same order of magnitude, although the Owens–Wendt approach returned slightly higher values in most of the film samples. This result could be a consequence of some differences between the theoretical definitions used by two methods [75].

Using the results of the surface energies, the work of adhesion and interfacial energy for each layer interface were calculated and are shown in Table 7. Both the Owens–Wendt and van Oss–Good approaches demonstrated the same tendencies in the work of adhesion and the interfacial energy.

Although the Biofilm and m-PVOH surfaces were characterized by very different SFE values (25.44 mN/m and 55.74 mN/m for Biofilm and Biofilm/m-PVOH samples, respectively), it is important to underline that a final good interlayer adhesion was achieved after the coating production, as was also confirmed by the SEM analyses and by the work of adhesion value, indicating suitable adhesion for polymer assembly at industrial scale [76]. This can be attributed to the presence of ethylenic aliphatic moieties in m-PVOH that interact with the non-polar groups of the Biofilm substrate, as confirmed by comparable values of the dispersive component for Biofilm and m-PVOH layers (23.73 and 31.96 mN/m, respectively). It is well known, indeed, that adhesion strength strictly depends on the amount of polar and dispersion bonds between the substrate and the coating [77] and the adhesion force is optimal when the *γ_s_^p^* and *γ_s_^d^* values are of the same order of magnitude.

It is also important to underline that both the coating layers are easily soluble and removable during post-consumer plastic-waste-separation operations, leaving unaltered the biodegradation profile of the substrate and opening a perspective for its mechanical recycling [78,79].

### 3.6. Evaluation of Seal Strength

Seal strength is an indicator of heat seal quality in flexible packaging, a key issue in the preservation of food quality and safety. According to the literature, there is a strong relationship among material properties and processing and adequate-uniform seal strength development [80].

**Figure 7:**
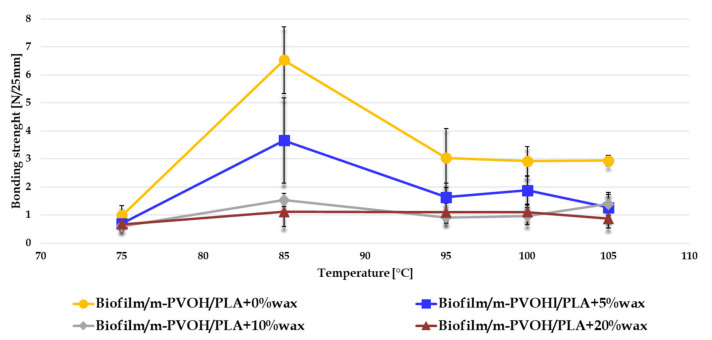
Bonding strength (N/25 mm) of the multilayer films.

To determine the seal strength, delamination tests were performed at temperatures within the range of 75–105 °C, as described in the “Experiment” section. Both the Biofilm and Biofilm/m-PVOH films were not sealable in this temperature range; the addition of the PLA coating layer therefore provided additional heat-sealable functionality to the structure, due to the good seal performances of the amorphous PLA [81,82]. The bonding strength, i.e., the maximum force required to peel or tear 25-milimeter-wide sealed samples, is reported in Figure 7.

All the multilayer films with up to 10% wax content exhibited the maximum bonding strength at a sealing temperature equal to 85 °C. Therefore, this temperature value can be considered the optimum sealing temperature for the double-coated systems.

A decrease in bonding strength values was observed upon increasing the wax loading. Characteristic sealing failures occurred through adhesive peeling at 75 °C; at higher temperatures, delamination failures in the PLA coating were more frequently observed. The mechanism of the deterioration of the adhesion performance can be attributed to a combination of chemical and physical interactions between the m-PVOH surface, the polylactic acid and the EBS wax. In particular, the micronized wax was responsible for changes in the morphology of the PLA matrix, as observed through the SEM analyses, which could have hampered the PLA molecules’ interactions and mobility, thus affecting adhesion at the sealing interfaces. By increasing the sealing temperature close to the melting temperature of the Biofilm substrate (above 110 °C), the bonding strength dramatically diminished, since the substrate deformation compromising its adhesion with the coating.

### 3.7. Tensile Properties

**Figure 8:**
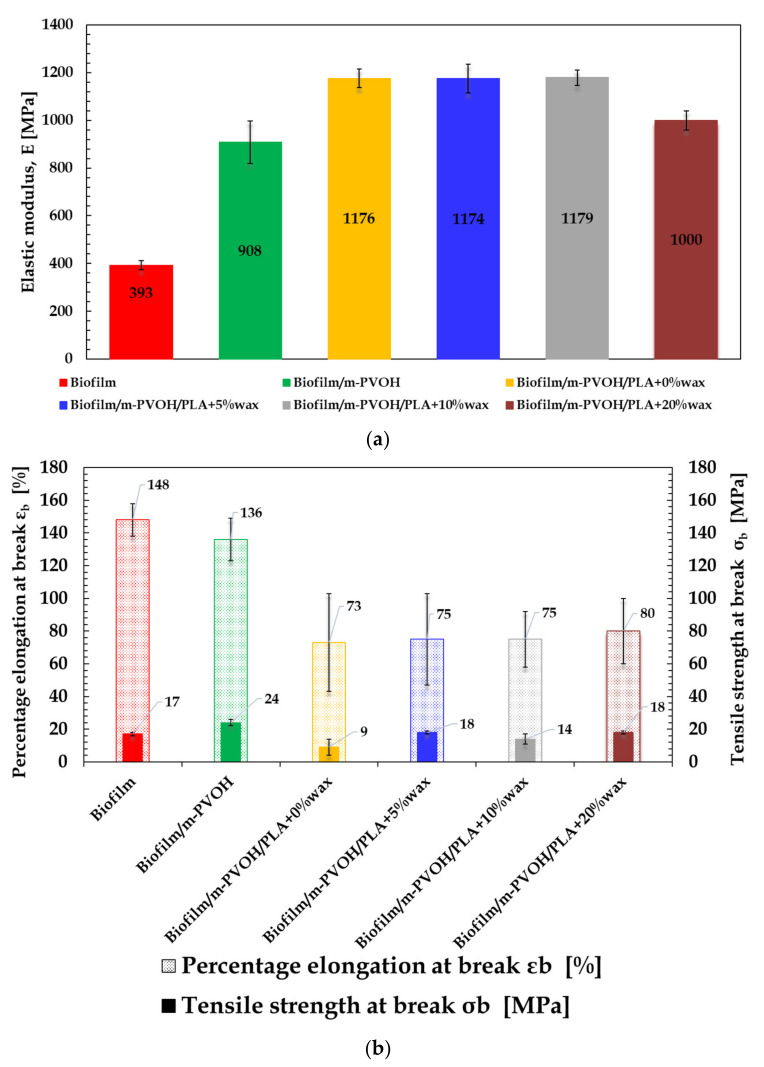
Elastic modulus (E) (**a**) and elongation at break (ε_b_) and tensile strength at break (σ_b_) (**b**) for pure Biofilm substrate and multilayer films.

In order to evaluate the effect of the addition of the coating layers on the mechanical performances of the substrate, as well as the influence of the wax’s incorporation, the tensile properties of the monolayer and multilayer films are compared, in the graphs in Figure 8, in terms of elastic modulus E, stress at break σ_b_ and strain at break ε_b_.

As expected, the mechanical properties of the Biofilm film were intermediate compared to those of PLA, which is rigid and brittle (E~2200 MPa, ε_b_~30% [83]), and PBAT, which isflexible and tough (E~120 MPa, ε_b_~500% [83]). In particular, the web layer exhibited good ductility, with a percentage of elongation at break equal to 148 ± 10% and other tensile parameters comparable to that of blends with PBAT content equal to 40% or higher [67,84].

The deposition of the m-PVOH coating over the Biofilm substrate led to an increase in the elastic modulus of +131% compared to the neat Biofilm. This outcome is in accordance with the literature data, which report E values for PVOH films within the range 2000–2300 MPa, depending on the polymer grade [85,86]. However, this did not substantially affect the sample ductility, as only a slight decrease (ca. 8%) in ε_b_ value occurred. The tensile strength at break, equal to 24 MPa, is comparable to those reported in the literature for neat PVOH resins [79].

The addition of the amorphous PLA4060D (E~1330 MPa, ε_b_~4% [69] as the second coating layer determined a further stiffening of the films, with the elastic modulus increasing up to 1176 ± 39 MPa and the elongation at break remaining above 73%. The addition of EBS up to 10% wax did not substantially affect the tensile performance with respect to the Biofilm/m-PVOH/PLA + 0% wax sample. Only a slight decrease in the elastic modulus and a slight increase in the elongation at break were detected for the PVOH/PLA + 20% wax system. The EBS, in fact, has a lubricant action and can promote the chain mobility of the PLA of the second coating layer [87].

## 4. Conclusions

In this study, novel biodegradable multifunctional films were produced by using the double-coating process and were characterized in terms of their microstructural, physical and functional properties, to determine their suitability as food packaging materials.

The infrared spectroscopy and thermal analyses revealed that the adopted coating procedure promoted the formation of intermolecular instead of intramolecular H-bonds within the polymer. This changed the structural and morphological state of the m-PVOH in the coating layer, which resulted in substantial amorphous behavior. The m-PVOH deposition led to a decrease in the oxygen transmission rate of three orders of magnitude, from 2200 cm^3^/m^2^ d bar, for the neat substrate, to 8.14 cm^3^ mm/m^2^ d bar. This result is particularly relevant with respect to other, comparable, biodegradable materials.

The deposition of the amorphous, PLA-based, second coating layer did not provide additional benefits for the barrier properties, while it determined a maximum bonding strength equal to 6.53 N/25 mm. EBS wax incorporation in the polymer matrix was responsible for some modifications in the PLA morphology, as highlighted by the SEM analyses. However, it was pointed out that, by increasing the wax concentration, the oxygen barrier and tensile properties were not significantly affected. On the other hand, the adhesion gradually decreased, while the surface hydrophobicity increased with an increase in the water contact angle from 65.4°, for the neat polylactide layer, up to 71° at 20% wax content. Future water permeability tests will be carried out on the produced films in order to further evaluate the effectiveness of the PLA + wax coating layers at hindering the vapor diffusion towards the sensitive central layer.

On the basis of the overall results, we demonstrated that the adopted double-coating strategy is a promising technique with which to combine the ductility and tear resistance of PBAT/PLA substrates with the excellent oxygen barrier properties of m-PVOH layers and the sealability and surface hydrophobicity provided by PLA + EBS wax layers.

In particular, the improvements in the O_2_ permeability made it possible to classify the produced systems as high-barrier packaging solutions, suitable for the packaging of O_2_-sensitive foods such as fresh meat and cheese. Of course, the definition of the target food depends on the balance of the overall functional performance, including the water vapor permeability, as well as the shelf-life requirements of the food.

## Figures and Tables

**Table 1:** List of the multilayer films produced with coating dry thickness measurements. The neat substrate (Biofilm) was used as a reference.

Sample Film	Wax Concentration (*w/w* PLA)	Thickness of the First Coating (μm)	Thickness of the Second Coating (μm)	Total Thickness (μm)
Biofilm	0	-	-	22 ± 2
Biofilm/m-PVOH	0	5 ± 1	-	27 ± 3
Biofilm/m-PVOH/PLA + 0% wax	0	5 ± 1	6 ± 1	33 ± 4
Biofilm/m-PVOH/PLA + 5% wax	5	5 ± 1	6 ± 1	33 ± 4
Biofilm/m-PVOH/PLA + 10% wax	10	5 ± 1	7 ± 2	34 ± 4
Biofilm/m-PVOH/PLA + 20% wax	20	5 ± 1	8 ± 2	35 ± 5

**Table 2:** Thermal parameters of the m-PVOH powder and m-PVOH thin-coating layer related to the first heating and cooling cycles.

Sample	First Heating				Cooling	
	T_g_ (°C)	DHm_rel_ (J/g)	T_m1_ (°C)	ΔH_m_ (J/g)	T_c_ (°C)	ΔH_c_ (J/g)
m-PVOH powder	42.7	1.8	215.4	70.4	150.7	45.2
m-PVOH coating	39.8	4.9	n.d.	n.d.	n.d.	n.d.

**Table 3:** Thermal parameters of the wax powder and PLA coating layers with 0%, 5%,10% and 20% of wax, related to the first heating and cooling cycles.

First Heating											Cooling	
Sample	T_g1_ (°C)	DHm_rel_ (J/g)	T_m1_ (°C)	DH_m1_ (J/g)	T_m2_ (°C)	DH_m2_ (J/g)	T_m3_ (°C)	DH_m3_ (J/g)	T_m4_ (°C)	DH_m4_ (J/g)	Tc1 (°C)	ΔHc1 (J/g)
Wax powder	n.d.	n.d.	69.5	7.7	87.7	2.1	104.4	8.5	146.7	119.8	140.8	121.7
PLA + 0% wax coating	60.1	3.4	n.d.	n.d.	n.d.	n.d.	n.d.	n.d.	n.d.	n.d.	n.d.	n.d.
PLA + 5% wax coating	60.8	4.2	n.d.	n.d.	n.d.	n.d.	109.6	3.03	145.9	6.84	127.0	7.3
PLA + 10% wax coating	59.4	2.5	n.d.	n.d.	n.d.	n.d.	110.7	3.96	145.8	9.99	132.1	10.7
PLA + 20% wax coating	60.1	3.6	n.d.	n.d.	n.d.	n.d.	110.2	7.7	145.6	20.0	139.5	20.4

**Table 4:** Oxygen transmission rate (OTR) for neat Biofilm and all multilayer films.

Sample Films	$\mathrm{OTR}\;\left[\frac{cm^{3}}{m^{2\;}d\;bar}\right]$
Biofilm	2200 ± 83.6
Biofilm/m-PVOH	8.14 ± 1.04
Biofilm/m-PVOH/PLA + 0% wax	10.0 ± 1.28
Biofilm/m-PVOH/PLA + 5% wax	9.39 ± 1.71
Biofilm/m-PVOH/PLA + 10% wax	9.41 ± 1.24
Biofilm/m-PVOH/PLA + 20% wax	8.58 ± 2.42

**Table 5:** Oxygen permeability of the m-PVOH single-coating layer, calculated in this study, and of other biodegradable materials (calculated values from Wu et al., 2021 [11]).

Sample Films	$P_{O2}\;\left[\frac{cm^{3}\;mm}{m^{2\;}d\;bar}\right]$
m-PVOH	0.047
PLA (semicrystalline)	3.36–15.0 (23 °C/50% or 0%)
PBAT	62.0 (23 °C/50%)
PBS	5.28 (23 °C/50%) 8.64 (20 °C/90%)
PHA	0.20 (23 °C/85%) 2.16 (23 °C/0%) 5.84 (25 °C/80%)
PCL	50.5 (25 °C/0%)

**Table 6:** Static/equilibrium contact angles for the different multilayers’ surfaces in water (H_2_O), diiodomethane (DM) and ethylene glycol (EG). Surface energies comprising polar and dispersive components and polarity following the Owens–Wendt approach; Lifshitz–van der Waals and acid–base components following the van Oss–Good approach.

Films Analysed Surface	Static Contact Angle (°)			Surface Energy (mN/m)/Polarity [−]							
				Disperse-Polar (Owens–Wendt)				Acid-Base (Van Oss–Good)			
	H_2_O	DM	EG	*γ_s_^p^*	*γ_s_^d^*	P_s_	*γ_S_*	*γ_s_^LW^*	*γ_s_* ^+^	*γ_s_* ^−^	*γ_S_*
Biofilm	97.9 ± 2	65.9 ± 2	80.2 ± 1	1.67	23.76	0.07	25.44	25.19	3.18	0.04	25.92
m-PVOH	47.4 ± 2	38.3 ± 1	39.8 ± 3	23.78	31.96	0.43	55.74	40.45	39.46	0.01	41.40
PLA + 0% wax	65.4 ± 1	37.5 ± 1	52.4 ± 1	11.63	35.07	0.25	46.70	40.84	21.31	0.10	43.76
PLA + 5% wax	67.2 ± 1	36.2 ± 1	52.3 ± 1	11.52	35.68	0.24	47.20	41.47	21.44	0.12	44.73
PLA + 10% wax	69.8 ± 1	37.0 ± 1	54.6 ± 1	10.72	35.56	0.23	46.27	41.09	20.67	0.18	44.95
PLA + 20% wax	71.0 ± 1	37.1 ± 2	56.6 ± 2	9.48	35.89	0.21	45.37	41.04	18.94	0.23	45.24

**Table 7:** Calculated work of adhesion and interfacial energy of the films’ interfaces following both the Owens–Wendt and van Oss–Chaudhury–Good approaches.

**Films Interface**	Owens-Wendt		Van Oss-Good	
	$W_{a}^{g}\;(\mathrm{mN}/m)$	$\gamma_{SL}^{g}\;(\mathrm{mN}/m)$	$W_{a}^{ab}\;(\mathrm{mN}/m)$	$\gamma_{SL}^{ab}\;(\mathrm{mN}/m)$
Biofilm/m-PVOH	67.7	13.4	66.7	0.6
m-PVOH/PLA + 0% wax	100.2	2.2	86.0	−0.8
m-PVOH/PLA + 5% wax	100.6	2.3	87.0	−0.9
m-PVOH/PLA + 10% wax	99.3	2.7	87.6	−1.2
m-PVOH/PLA + 20% wax	97.8	3.3	88.2	−1.6
